# Fatal asthma; is it still an epidemic?

**DOI:** 10.1186/s40413-016-0129-9

**Published:** 2016-12-14

**Authors:** Andrea Vianello, Marco Caminati, Mariangiola Crivellaro, Rafi El Mazloum, Rossella Snenghi, Michele Schiappoli, Annarita Dama, Andrea Rossi, Giuliana Festi, Maria Rita Marchi, Chiara Bovo, Giorgio Walter Canonica, Gianenrico Senna

**Affiliations:** 1 Respiratory Pathophysiology Division, University-City Hospital of Padua, Padua, Italy; 2 Asthma Center and Allergy Unit, Verona University and General Hospital, piazzale Stefani 1, 37126 Verona, Italy; 3 Allergy Service, Department of Medicine and Public Health, University of Padua, Padua, Italy; 4 Department of Cardiac, Thoracic and Vascular Sciences, University of Padua, Padua, Italy; 5 Medical Direction, Verona University and General Hospital, Verona, Italy; 6 Allergy and Respiratory Diseases, IRCCS San Martino-IST, University of Genoa, Genoa, Italy

**Keywords:** Fatal asthma, Asthma mortality, Asthma exacerbations, Alternaria

## Abstract

**Background:**

Asthma mortality has declined since the 1980s. Nevertheless the World Health Organization (WHO) identified asthma as responsible for 225.000 deaths worldwide in 2005, and 430.000 fatal cases are expected by 2030. Some unexpected and concentrated fatal asthma events all occurred between 2013 and 2015 in Veneto, a North Eastern region of Italy, which prompted a more in-depth investigation of characteristics and risk factors.

**Methods:**

A web search including key words related to fatal asthma in Italy between 2013 and 2015 has been performed. Concerning the cases that occurred in Veneto, subjects’ clinical records have been evaluated and details about concomitant weather conditions, pollutants and pollen count have been collected.

**Results:**

Twenty-three cases of asthma deaths were found in Italy; 16 of them (69%) occurred in the Veneto Region. A prevalence of male and young age was observed. Most of patients were atopic, died in the night-time hours and during the weekends. The possible risk factors identified were the sensitization to *alternaria*, previous near fatal asthma attacks and the incorrect treatment of the disease. Weather condition did not appear to be related to the fatal exacerbations, whereas among the pollutants only ozone was detected over the accepted limits. Smoking habits, possible drug abuse and concomitant complementary therapies might be regarded as further risk factors.

**Discussion:**

Although not free from potential biases, our web search and further investigations highlight an increasing asthma mortality trend, similarly to what other observatories report. The analysis of available clinical data suggests that the lack of treatment more than a severe asthma phenotype characterizes the fatal events.

**Conclusions:**

Asthma mortality still represents a critical issue in the management of the disease, particularly in youngsters. Once more the inadequate treatment and the lack of adherence seem to be not only related to the uncontrolled asthma but also to asthma mortality.

## Background

**Fig. 1:**
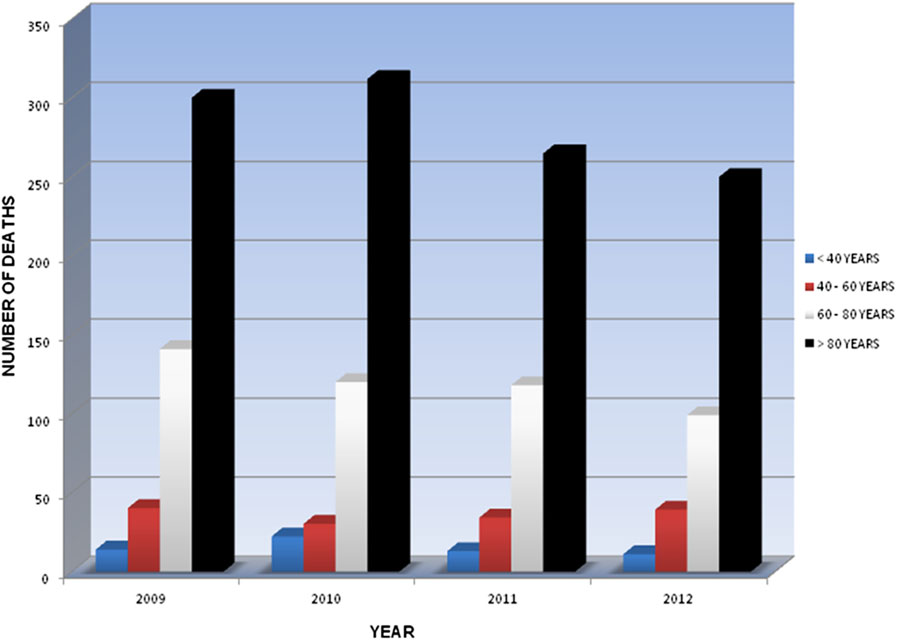
Trend of asthma mortality according to the Italian National Institute of Statistics (ISTAT) from 2009 to 2012

Asthma mortality declined since the 1980s, at least in Europe and in the United States [1–4]. The widespread use of inhaled steroids probably accounts for it [5]. Nevertheless the World Health Organization (WHO) identified fatal asthma as responsible for 225.000 deaths worldwide in 2005 and, according to the current trend, for 430.000 by 2030 [6, 7]. Also in Italy, a reduction in asthma deaths was registered in the period 2009–12 [8], and most of recorded cases were elderly (>60 years) (Fig. 1). The prevalence of fatal asthma in older age might be related to the inaccuracy of certification procedures. In fact, several studies have proved an overestimation of asthma mortality in the elderly, with a degree of inaccuracy in certification ranging from 39 to 80% [9, 10]. The incorrect identification of Chronic Obstructive Pulmonary Disease (COPD) instead of asthma as the cause of death appears to be responsible for many wrong certifications. In fact the terms “asthma” and “COPD” have come to be used interchangeably, because both conditions share the airflow obstruction as well as similar medications [11]. Furthermore the presence and severity of comorbidities might hamper the identification of the main cause of death in elderly asthmatics patients. On the contrary near-fatal or fatal asthma seems to be partly restricted to youngsters or adults below 50 years and under the epidemiological perspective, they can be considered as part of the same disease cluster [12, 13]. In a recent study evaluating a large series of patients affected by near-fatal asthma (NFA), the majority of them were youngsters or adults below 50 years [14]. However, despite its relevance in terms of epidemiology and emotional impact, fatal asthma is not yet fully understood and preventable. In fact on a clinical ground it shows a very heterogeneous phenotype, characterized by variable causes and risk factors. Thus asthma mortality still represents a critical issue in the management of the disease.

Three unexpected cases of fatal asthma occurred in Padua (a town in the North East of Italy) in July 2015. This was the starting point for a more extensive evaluation of fatal asthma in our region. We aimed at analyzing asthma deaths that occurred in the last 3 years (2012–2015) in the Veneto region, in the North East of Italy, in order to identify and highlight characteristics and risk factors of fatal asthma.

## Methods

Acknowledging the intrinsic flaws of the official data produced by ISTAT, the Italian National Institute of Statistics [8], the identification of cases and circumstances of asthma deaths was carried out in three different steps.

- In order to detect recent cases of fatal asthma in Italy a web search was performed using Google™. The following key words were included: [fatal asthma], [asthma death], [asthma mortality]. The time frame ranged from 2013 to 2015. In fact, a longer interval might negatively affect the availability and reliability of clinical data.
- Concerning the cases occurring in Veneto Region, a more in-depth investigation was carried out.
  i. The full newspaper articles were collected. Data about circumstances of death, such as date and place, as well as age and gender of cases were extrapolated.
  ii. Subjects’ clinical records were searched at the Emergency Departments of the Hospitals where the patients had been admitted or among the medical files of their GPs.
  iii. Information concerning the weather conditions (temperature, humidity, rainfall, thunderstorm), the levels of pollutants (O_3_, PM_10_, NO_2_) and the pollen count at the time and at the place of the fatal attack were collected from the website of the regional Agency for the Environment Prevention and Protection (ARPA), www.arpa.veneto.it [15].

## Results

Twenty-four cases of asthma deaths were found in Italy within the indicated time frame (2013–2015); 17 of them (71%) occurred in the Veneto Region.

Demographic data are shown in Table 1. Three out of 17 subjects were female (17.6%) and the mean age was 26.1 years (range 11–51). However most of the patients were under 41 years old: 35% were between 10 and 20 years old, 53% between 21 and 40 years old and the other ones were older. According to the region of origin, most of patients were Italian (71%) whereas four subjects were immigrants, coming respectively from Russia, Romania, Morocco and Philippines. Seven patients were students; among the adults three of them were unemployed.

**Table 1:** Demographic data of patients and place and time of fatal events

Patient’s initials	Place (county)	Age	Gender	Nationality	Time of death	Occupation	Place of death	Day of the week
GP	Padua	18	M	Italy	1 AM	student	Outdoor	FRI
JV	Padua	16	M	Italy	8 PM	student	At home	WED
LM	Treviso	22	M	Italy	3 AM	mechanic	Hospital	THU
SM	Venice	22	M	Italy	10 PM	chef	Hospital	FRI
RB	Verona	10	M	Italy	5 PM	student	Hospital	MON
ST	Padua	15	M	Italy	8 PM	student	At home	FRI
AR	Verona	34	M	Italy	2 AM	employee	At home	SAT
SH	Padua	26	M	Morocco	10 PM	unemployeed	At home	SAT
MP	Padua	33	F	Italy	8.30 PM	painter	Outdoor	MON
AV	Vicenza	11	M	Italy	11.30 PM	student	At home	FRI
KS	Treviso	21	M	Romania	4 AM	employee	At home	WED
EA	Padua	42	F	Philippines	7 AM	unemployeed	At home	FRI
KK	Venice	54	M	Russia	7 PM	employee	Train station	SUN
RT	Padua	18	M	Italy	11 PM	student	At home	SAT
MA	Padua	30	M	Italy	9 PM	student	At home	FRI
OZ	Padua	41	M	Morocco	3 AM	unemployeed	Outdoor	SAT
AB	Padua	31	F	Italy	3 AM	journalist	At home	WED

Overall, patients died at home (59%) and only three patients reached the Emergency Room. Three cases died during outdoor activities. Ten patients had the fatal attacks during the weekend (59%) and the decease occurred in the evening or during nighttime. The temporal distribution of the deaths over the year showed a cluster between June and October, when nine patients died. Seven patients died between November and March.

Considering the weather condition at the place and time of the fatal attacks, the temperature was in line with the seasonal average values, rainfalls generally were absent and no thunderstorms were registered (Table 2).

**Table 2:** Weather conditions at the place and time of fatal attacks

Patient’s initials	Place (County)	Date	Time	Temperature (°C)	Rainfall	Thunderstorm	Place of death	Day of the week
GP	Padua	01.11.13	1 AM	11°	Absent	Absent	Outdoor	FRI
JV	Padua	07.01.14	8 PM	5°	Absent	Absent	At home	WED
LM	Treviso	27.02.14	3 AM	8°	Absent	Absent	Hospital	THU
SM	Venice	14.03.14	10 PM	10°	Absent	Absent	Hospital	FRI
RB	Verona	16.06.14	5 PM	16°	Absent	Absent	Hospital	MON
ST	Padua	08.08.14	8 PM	24°	Absent	Absent	At home	FRI
AR	Verona	30.08.14	2 AM	22°	Absent	Absent	At home	SAT
SH	Padua	13.09.14	10 PM	19°	1 mm	Absent	At home	SAT
MP	Padua	29.09.14	8.30 PM	17°	Absent	Absent	Outdoor	MON
AV	Vicenza	17.10.14	11.30 PM	18°	Absent	Absent	At home	FRI
KS	Treviso	12.11.14	4 AM	12°	Absent	Absent	At home	WED
EA	Padua	19.12.14	7 AM	6°	Absent	Absent	At home	FRI
KK	Venice	01.03.15	7 PM	9°	Absent	Absent	Train station	SUN
RT	Padua	11.07.15	11 PM	23°	Absent	Absent	At home	SAT
MA	Padua	18.07.15	9 PM	29°	Absent	Absent	At home	FRI
OZ	Padua	19.07.15	3 AM	26°	Absent	Absent	Outdoor	SAT
AB	Padua	05.11.15	3 AM	12°	Absent	Absent	At home	WED

**Fig. 2:**
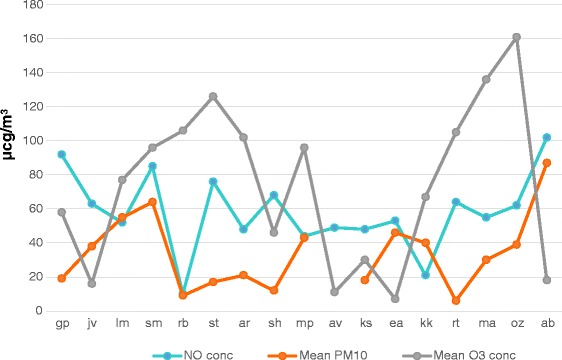
Levels of pollutants (NO, PM_10_ and O_3_) registered at the place and at the time of the deaths. On x-axis patients’ initials are reported. In 3 days the concentration of ozone was above the accepted limits (120 μcg/m^3^), whereas only in 1 day the level of PM_10_ was significantly over the permitted values (50 μcg/m^3^)

Regarding airborne pollutants, in four cases an increase of PM10 (one case, AB) and O3 (three cases, ST, MA and OZ) significantly over the accepted values was registered in concomitance with the fatal attacks (Fig. 2).

**Fig. 3:**
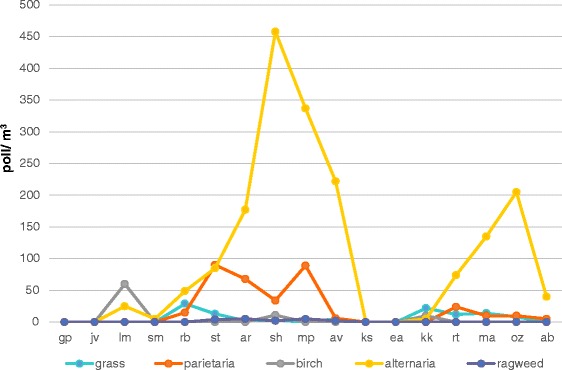
Aerobiological data at the time and places of the deaths are shown. On x-axis patients’ initials are reported. The pollen count was low for all pollens detected (grass, birch, parietaria and ragweed) whereas in 6 days a high concentration of alternaria was registered

Figure 3 shows the pollen count data at the time and place of the events. An Alternaria peak was registered in six cases (AR, SH, MP, AV, MA, OZ) and a medium concentration was detected in concomitance with three other fatal attacks (RB, ST, RT). A 3 days long lasting Parietaria increase was reported in three cases (ST, AR, MP).

**Fig. 4:**
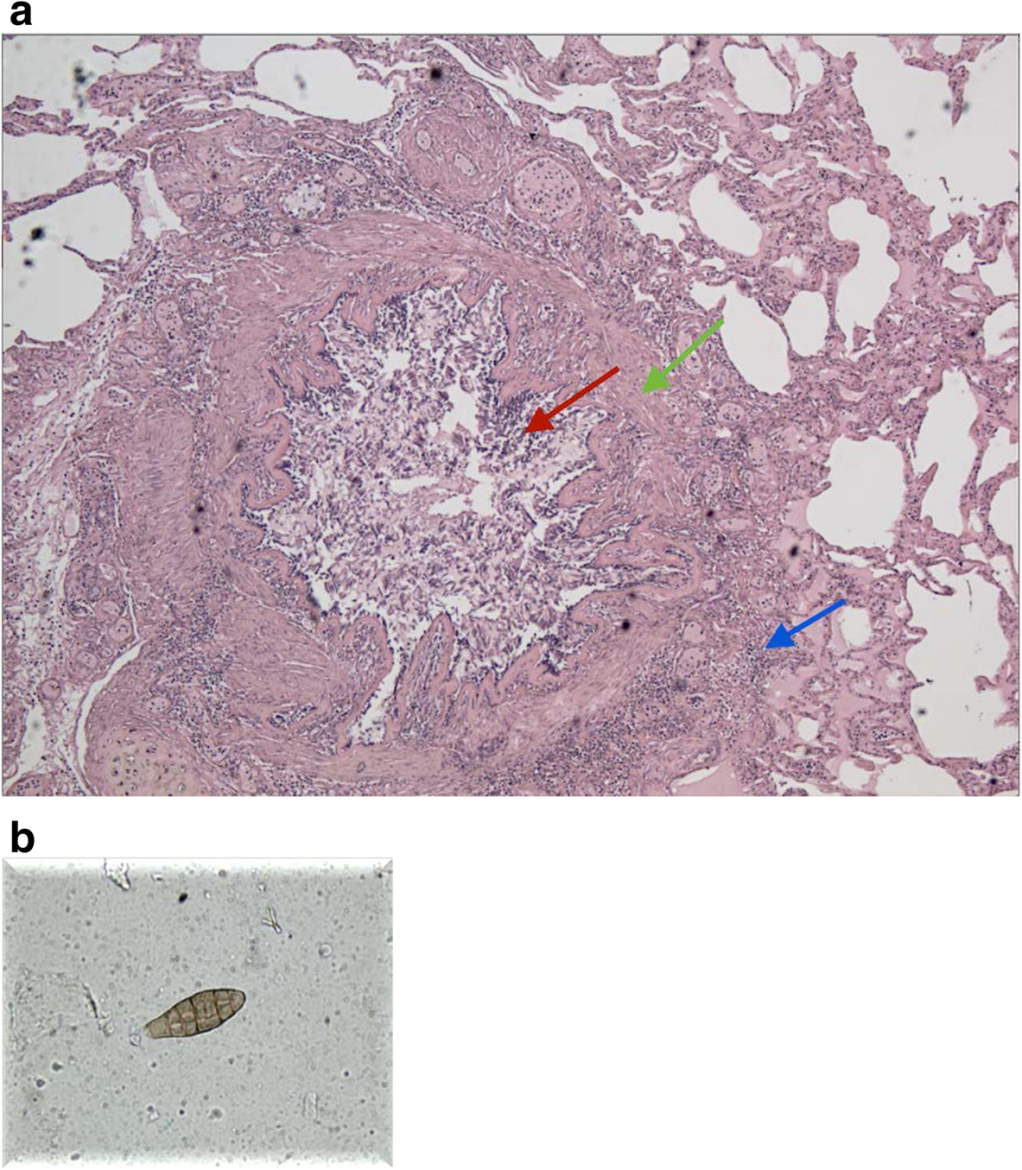
A massive infiltration and airways obstruction has been detected in the airways of a patient who died during the summer (**a**). The image shows a section of bronchus - hematoxylin-eosin stain - magnification × 100. *Red arrow*: bronchial lumen occluded by amorphous eosinophilic material corresponding to mucus plugs, mixed with inflammatory cells and epithelial cells. *Green arrow*: peribronchial and sub-mucosal hyperaemia, thickening of the basal membrane with muscle hypertrophy. *Blue arrow*: neutrophilic and limphomonocitary inflammation. The patient was sensitized to alternaria, which was detected in the airways (**b**); the outdoor concentration of the fungal mold was high at the time of the death

Complete clinical details were obtained for eight patients, partial details for five, whereas in the remaining four cases the diagnosis was confirmed by the autopsy but the preexisting clinical data were lacking (Table 3). According to the available clinical data, all the patients were atopic. Grass pollens, mite and Alternaria were the most common sensitizers. None of the patients was on regular treatment, but according to the medical files, 13 patients were advised to take short acting beta agonist (SABA) as needed and irregularly courses of inhaled corticosteroids (ICS) alone or in combination with long acting beta agonists. Smoking habit, food allergy, drug abuse, physical exercise, concomitant use of complementary therapies were reported as potential risk factors in several cases. Three patients were repeatedly admitted to ER before the fatal attack and were treated with epinephrine and oxygen supplies. In 14 cases the autopsy confirmed the diagnosis. In one subject (MP), besides the massive lymphocytic and eosinophilic inflammatory infiltrate occluding the bronchial lumen, alternaria was detected in the airways together with mucus plugs (Fig. 4). In two cases the autopsy was not performed, according to the certainty of the clinical diagnosis.

**Table 3:** Clinical features of patients suffering from fatal asthma

Patient’s initials	Atopy	Follow-up	Concomitant risk factors	Allergic sensitizations	Asthma treatment	History of Hospital/ER admissions
GP	Present	infrequent	Smoking, party, physical exercise	NR	Intermittent use fo SABA	any
JV	Present	NR	Physical exercise	NR	Intermittent use fo SABA	Any
LM	Present	Infrequent	Heavy smoker	Grass, mites, peach	Intermittent use fo SABA	In childhood 3 years in an high altitude hospital, recently several admission to ER for asthma exacerbations
SM	Present	Infrequent	Smoking	Mites	SABA as needed	Admission to ER for asthma exacerbation; treated with epinephrine and Oxigen supply
RB	Present	infrequent	Outdoor physical exercise, concomitant use of homeopathic remedies	Mites, Parietaria, grass	Intermittent use of SABA and short courses of ICS	Any
ST	Present	regular	Severe asthma in childhood	Mites, grass, alternaria	SABA as needed. Short courses of ICS	Any
AR	Present	infrequent	Dinner	Grass, mites alternaria	Intermittent use of ICS-LABA and SABA as needed	Any
SH	NR	NR	NR	NR	NR	NR
MP	Present	infrequent	NR	Grass, Alternaria	ICS-LABA, short courses of oral steroids	Any
AV	Present	regular	Physical exercise, hours spent outdoor	Grass, Alternaria, Mites	SABA as needed. Intermittent use of ICS	Any
KS	NR	NR	NR	NR	NR	NR
EA	NR	NR	NR	NR	NR	NR
KK	NR	NR	NR	NR°	NR	NR
RT	Present	2	Food allergy	Grass, parietaria, mites	SABA as needed, short courses of ICS	Any
MA	present	2	NR	Mites, Alternaria°	SABA as needed	Any
OZ	present	4	Drug abuse, heavy smoker	Referred mites	SABA as needed	Many admissions to ER
AB	present	Infrequent	NR	Mites	SABA as needed	Any

*NR* not reported

## Discussion

Seventeen cases of fatal asthma, all occurring in the same Italian region within the last 3 years, have been detected and analyzed. A prevalence of male gender, young age and atopic condition was observed. Several risk factors have been identified such as sensitization to *Alternaria*, previous near fatal asthma attacks, incorrect treatment of the disease. Weather condition or thunderstorms did not trigger the fatal exacerbation, whereas among the pollutants only ozone was detected over the accepted limits during the days when the deaths occurred.

We observed an increased asthma rate in young males, much younger then in ISTAT asthma deaths database [8], compared to young females [16]. It may be related to the prevalence of male gender among the described cases. However, the prevalence of male gender reflects what is known in the general population [8]. Most of subjects (44%) were students whereas three adult patients were unemployed. The last were all immigrants, suggesting difficult access to the health care resources as a potential risk factor for uncontrolled asthma. In fact, one patient had been admitted to the Emergency Room several hours before his death, but voluntarily he went away from the waiting room. Four hours later he was found dead in a street nearby, with the inhaler in his hand. The other two cases had the fatal attack at home, but they had not been visited by a doctor during the last years. The autopsy proved the causal role of asthma in all these fatal cases. This finding is in agreement with the higher mortality noticed in populations with lower income, such as black or Hispanic in the United States. However, differently from economical issues, racial factors at least in youngsters seemed not to affect asthma severity [17]. Of note, according to a large USA study that investigated the outcomes of patients hospitalized for asthma, race was not relevant as a risk factor for hospital deaths. Caucasian patients show an overall lower risk of death in comparison with black patients, but factors preceding hospitalization, such as lower income, do probably account for it [18].

The cluster of fatal attacks during the weekend emphasizes the potential role of smoking, drug or alcohol abuse, which are quite common during parties or other entertainment among youngsters, as negative cofactors in determining the decease. Smoking habit is still common in young people. All the studies including adolescents reported higher rates of smoking in asthmatics than in non-asthmatic peers, though this trend is less consistent in adults [19]. Moreover, an increased prevalence of asthma and COPD has been reported in patients who smoke crack or cocaine [20].

In our series, most of the fatal cases occurred in the evening and during the night. It is well known that nocturnal exacerbations occur in 2/3 of asthmatics, contributing to the morbidity and mortality of the disease [21]. Several factors might account for this finding, such as changes in respiratory function related to sleep as well as processes related to circadian regulation. Furthermore, concomitant diseases, such as gastro-esophageal reflux or sleep apnea may negatively affect the airway resistance overnight [21].

Weather conditions seemed not to be related to fatal attacks, as temperatures were in line with the average values of the season and there was no rainfall or thunderstorm in the critical days (Table 2). Only ozone was over the permitted threshold for 3 days. It has been shown that this pollutant is responsible for airway inflammation, as it increases the pro-inflammatory markers and oxidative stress in bronchial epithelial cells [22]. This finding was mainly observed in atopic individuals, in children as well as in adults [22, 23]. In our series one of the fatal events occurred in concomitance with a high level of ozone. He was atopic and a heavy smoker and it is possible that the pollution might have played a negative role by increasing airways inflammation.

Regarding the aerobiological data, the high concentration of *Alternaria* spores, with an overall low pollen count, also suggests a relevant role of this allergenic source [12]. The sensitization to this fungal mould was confirmed in five patients and maybe related to the fatal events. Moreover in one patient, besides the massive lymphocytic and eosinophilic inflammation in the bronchial airways, *Alternaria* was detected in the airways (Fig. 4). As previously described [24], the inflammatory reaction triggered by an allergen is responsible for a massive production of mucus and a decrease of the airway clearance function. Once the mucus plugging becomes extensive, even a slight bronchial muscle constriction on top of the plugs can cause the complete airway occlusion. Since 1991 *Alternaria* has been reported as responsible for NFA and fatal asthma [25], and epidemic asthma in the late summer and autumn was repeatedly reported with a peak of multiple hospital admissions in young asthmatics [26–28]. Several mechanisms may account for alternaria-induced exacerbations. In fact, besides the IgE mediated allergic inflammation, an *Alternaria*-specific serine protease activity is responsible for an immediate release of IL-33, which drives a robust Th2 inflammation and exacerbation of allergic airway disease [29]. Furthermore, the *Alternaria* induced IL-33 production can provoke a steroid resistance, as in children sensitized to this fungal mould a relationship between levels of IL-33 and use of oral steroids has been demonstrated [30]. In our series most of the patients were atopic, although not in all the cases the sensitization could possibly account for the death. Nevertheless, several patients of our series seemed to belong to a cluster of a recently identified NFA phenotype, whose features are the younger age, the smoking habit and the sensitization to *Alternaria* [14].

Respiratory infections might account for the cases of fatal asthma registered in winter [31], but this connection cannot be confirmed according to the clinical files of our cases.

In our case series, three patients were previously admitted to Emergency Rooms or Hospitals for near-fatal asthma (respiratory failure with hypercapnia). These patients, who can be classified as severe asthmatics according to the European Respiratory Society (ERS) Guidelines [32], indirectly confirm that severe asthma exacerbations remain a robust predictive risk factor for asthma mortality [33–35]. In the other patients the identification of the level of asthma severity is not easy. In fact according to the concomitant treatment reported in medical files, patients were affected by GINA level 2 or 3 [36], but detailed data about the concomitant asthma control are lacking. According to the UK National Review of Asthma Deaths [35], the largest study worldwide investigating asthma deaths, only 39% of patients were affected by severe asthma at the time of death. The other subjects suffered from mild to moderate asthma. Those findings suggest that undertreated and poorly controlled asthma rather than severe asthma is at high risk of fatal exacerbation.

Among the cases we have investigated, only one patient was regularly monitored, whereas the remaining cases were not regularly followed-up by their GPs. The lack of specialist and general practitioner supervision during the 12 months prior to death also characterizes the majority of fatal cases reported in the UK National Review of Asthma Deaths. As a probable consequence, a minority of patients was provided with a personal action plan and 45% of people seemed not to seek medical assistance before death [35].

All the described subjects shared the use of SABA as needed and only intermittent courses of ICS alone or in combination with LABA. This finding suggests that all the patients were using a rescue treatment but they did not follow a regular anti-inflammatory action plan. Moreover the underuse of ICS in comparison with SABA, confirmed by the GPs prescription in seven cases, suggests a low adherence to the anti-inflammatory treatment. Recently in patients with mild asthma the excessive use of SABA was identified as a reliable marker of poor control and increased risk of severe exacerbation [33, 34]. The over-prescription of SABA and the under-prescription of preventer medication have been described as a key finding of the UK National Review of Asthma Deaths [35]. Inappropriate prescribing of long-acting beta agonists was also highlighted: 14% of fatal asthma cases were prescribed a single-component bronchodilator, without any inhaled corticosteroid preventer treatment. However, the treatment of life-threatening asthma during the acute phase still represents a challenge. Some authors have suggested the use of epinephrine intramuscular auto-injector as an extreme emergency treatment able to save lives [37].

In our case series, for one patient the concomitant use of complementary medicine was reported. The intake of herbal or homeopathic remedies has been reported as a risk factor for uncontrolled asthma, as patients reduce or stop the pharmacological treatment in favor of the complementary treatment [38].

One major flaw of this survey is the method used for searching fatal asthma cases. The prevalence of patients below 40 years may be due to a “mediatic bias” since deaths at younger ages are more striking and more easily reported. Given this limitation, the identification of causes and risk factors can be more understandable at younger ages for the lack of relevant concomitant diseases, which are common in elderly and can be a confounding factor.

## Conclusions

Asthma mortality still represents a critical issue in the management of the disease. Our case series, prompted by some unexpected and concentrated fatal asthma events, lead to a more in-depth investigation of characteristics and risk factors. Surprisingly, a young age of the fatal asthma cases was found. The association between *Alternaria* sensitization and asthma mortality is proved in our study and suggests that asthmatics allergic to this mould have to be carefully monitored during summer and autumn and regularly treated. In our series, a high level of ozone in the environment and the smoking habit could have had a negative impact on the fatal attack. Finally, once more the inadequate treatment and the lack of adherence seem to be not only related to the uncontrolled asthma but also to asthma mortality.

## Abbreviation

NFA: Near-fatal asthma
